# Environmental Driving of Adaptation Mechanism on Rumen Microorganisms of Sheep Based on Metagenomics and Metabolomics Data Analysis

**DOI:** 10.3390/ijms252010957

**Published:** 2024-10-11

**Authors:** Haiying He, Chao Fang, Lingling Liu, Mingming Li, Wujun Liu

**Affiliations:** 1 Department of Animal Science and Biotechnology, Xinjiang Agricultural University, Urumqi 830052, China; 15733286826@163.com (H.H.); linglingliu1988@xjau.edu.cn (L.L.); 17699430630@163.com (M.L.); 2 Faculte de Medecine Veterinaire, Universite de Liege, Quartier Vallee 2, Avenue de Cureghem 6 (B43), 4000 Liege, Belgium; chao.fang@doct.uliege.be

**Keywords:** sheep, rumen, driving adaptations, metagenomics, metabolomics

## Abstract

Natural or artificial selection causes animals to adapt to their environment. The adaptive changes generated by the rumen population and metabolism form the basis of ruminant evolution. In particular, the adaptive drive for environmental adaptation reflects the high-quality traits of sheep that have migrated from other places or have been distant from their origins for a long time. The Hu sheep is the most representative sheep breed in the humid and low-altitude environments (Tai Lake region) in East Asia and has been widely introduced into the arid and high-altitude environments (Tibetan Plateau and Hotan region), resulting in environmental adaptive changes in the Hu sheep. In this study, a joint analysis of the rumen microbial metagenome and metabolome was conducted on Hu sheep from different regions (area of origin and area of introduction) with the objective of investigating the quality traits of Hu sheep and identifying microorganisms that influence the adaptive drive of ruminants. The results demonstrated that the growth performance of Hu sheep was altered due to changes in rumen tissue and metabolism following their introduction to the arid area at relatively high altitude. Metagenomic and metabolomic analyses (five ramsper area) revealed that 3580 different microorganisms and 732 different metabolites were identified in the rumen fluid of arid sheep. Among these, the representative upregulated metabolites were 4,6-isocanedione, methanesulfonic acid and N2-succinyl-L-arginine, while the dominant microorganism was *Prevotella ruminicola*. The downregulated metabolites were identified as campesterol, teprenone and dihydroclavaminic acid, while the disadvantaged microorganisms were *Dialister_succinatiphilus*, *Prevotella_sp._AGR2160*, *Prevotella_multisaccharivorax* and *Selenomonas_bovis*. The results of the Pearson analysis indicated that the rumen microbiota and metabolite content of sheep were significantly altered and highly correlated following their relocation from a humid lowland to an arid upland. In particular, the observed changes in rumen microorganisms led to an acceleration of body metabolism, rendering sheep highly adaptable to environmental stress. *Prevotella_ruminicola* was identified as playing an important role in this process. These findings provide insights into the environmental adaptation mechanisms of sheep.

## 1. Introduction

In addition to the factors of artificial selection, the most important factor in animal evolution is the driving force of environmental factors on the adaptability of animals [1]. Populations that have lived for many generations in environments that are different from those of the original species (from low to high, warm to cold or wet to dry), or in extreme environments, have been under selective pressure and have become physiologically and genetically adapted to live in those environments. Environments shape the genetic landscape of the populations that inhabit them [2]. Different aspects of the biological population have corresponding driving adaptations, such as growth and development [3,4], respiratory system [5,6], digestive system [7,8], etc. Animal genetic resources are the foundation for the sustainable development of the livestock industry and are vital to food security and the livelihoods of millions of people [9]. Since their domestication, sheep (*Ovis aries*) have provided humans with meat, wool, skin, milk and other products [10]. Since their domestication from the Asian mouflon in the Middle East some 10,000 years ago, sheep have spread to other parts of the world. Through adaptation to a wide range of environments and genetic improvement under different production systems, sheep have achieved a global distribution and developed into many unique breeds [11]. As they spread over the following centuries, mutations and natural and artificial selection occurred. This has led to different populations adapting to different environments in a process of biodiversification.

Gastrointestinal adaptations play an important role in animal energetics, as the portal venous viscera account for 20% of total oxygen consumption in ruminants [12]. The most important feature of ruminants is the highly developed rumen system compared to other mammals. The rumen is the hallmark organ of ruminants and hosts a diverse ecosystem of microorganisms that facilitate efficient digestion of plant fiber [13]. Microbiomes (known as ‘second genomes’) are closely linked to nutrient uptake, adaptability and host health [14]. The rumen microbiota play an important role in maintaining host metabolism [15], and the metabolic activity of the rumen microbiota can be modified by numerous factors derived from the host, the host environment and the microbiota itself [16]. Ruminants require constant adaptation to environmental changes to adapt to different ecological niches and feeding habits. In addition, the morphology and function of the ruminant digestive systems reveal some adaptive evolutionary features [17]. To adapt to the harsh environment of extreme cold, high altitude and hypoxia, yaks living on the Tibetan Plateau have raised many questions about the function and dynamics of the rumen microbiota [18]. As a typical mountain ruminant, the blue sheep adapts to the environment by eating and excreting rapidly [17]. With the development of sequencing technology, second-generation sequencing has played an important role in the study of the role of gut microorganisms. A total of 336 organisms have been discovered in available rumen metagenomic datasets by the Global Research Alliance’s Livestock Research Group [19]. It has been reported that metagenomic sequencing has been used to reveal the classification and functional characteristics of goat rumen microorganisms. In addition, the strong redundancy among the major functions in the rumen ecosystem limits the potential number of unique and specific relationships between microbial species and functional capabilities [20]. Legumes and salt bushes are potential alternatives to conventional fodder to overcome fodder shortages in arid and semi-arid countries. To adapt to the arid environment, camels have a specific rumen microbiota, Bacteroidetes and Firmicutes were the main bacterial phyla, and the main genera were *Prevotella*, *RC9_gut_group* and *Butyrivibrio*, which were over-represented in non-extracted plants [21]. It has been reported that 16 different genera of rumen microorganisms have been screened in camels for adaptation to the arid environment [22]. Batinah goats cope with drought and water scarcity, which increases the concentration of rumen fungi [23]. All of this suggests that rumen microbial communities can be used to study the response of ruminants to environmental drought stress. It has been reported that the rumen microbiome may be a reservoir of antibiotic resistance genes. *Ruminal Escherichia coli* within the Proteobacteria phylum was the main carrier of antibiotic resistance genes (ARGs) in goats consuming colostrum, while *Prevotella ruminicola* and *Fibrobacter succinogenes*, associated with cellulose degradation, were the carriers of ARGs after starter supplementation [24]. This also implies a close relationship between rumen microorganisms and antibiotics, suggesting that molecular markers can be identified for breeding to adapt to drought environments from this perspective. As sequencing technology develops and costs decrease, more and more gut microbial genomes will be revealed, improving the survival rate of the population and ensuring the safety of livestock products.

Despite the management challenges associated with the need for greater adaptation, several domesticated ruminants have also been successfully introduced to the alpine pastures for meat and dairy production, such as hybrid sheep, goats and cattle [25,26,27]. The origin of the Hu sheep is the Taihu region of China, which is humid and low-lying, and the Hu sheep have high fertility. As a result, Hu sheep have been introduced throughout China, helping to improve the breeding ability of sheep. Sheep farming is a traditional industry in Xinjiang. Modern grassland animal husbandry, under the premise of ecological and environmental protection, requires more livestock to be raised in agricultural areas. Due to the high cost of farmland, breeders are more willing to select high-fertility breeds to improve the economic benefits of breeding. From 2017, Xinjiang has introduced 30,000 Hu sheep every year to solve the problem of an insufficient mutton market. However, the arid climate of the desert in southern Xinjiang poses a great challenge to the introduction of Hu sheep, and the average survival rate of Hu sheep in many large-scale sheep farms is only 50%. To this end, the differences in rumen microorganisms and metabolites between Hu sheep in the Hotan area of Xinjiang and native Hu sheep were analyzed to reveal the adaptive characteristics and mechanisms of Hu sheep under extreme environmental pressure of drought.

## 2. Results

### 2.1. Characterization of Phenotype

**Figure 1:**
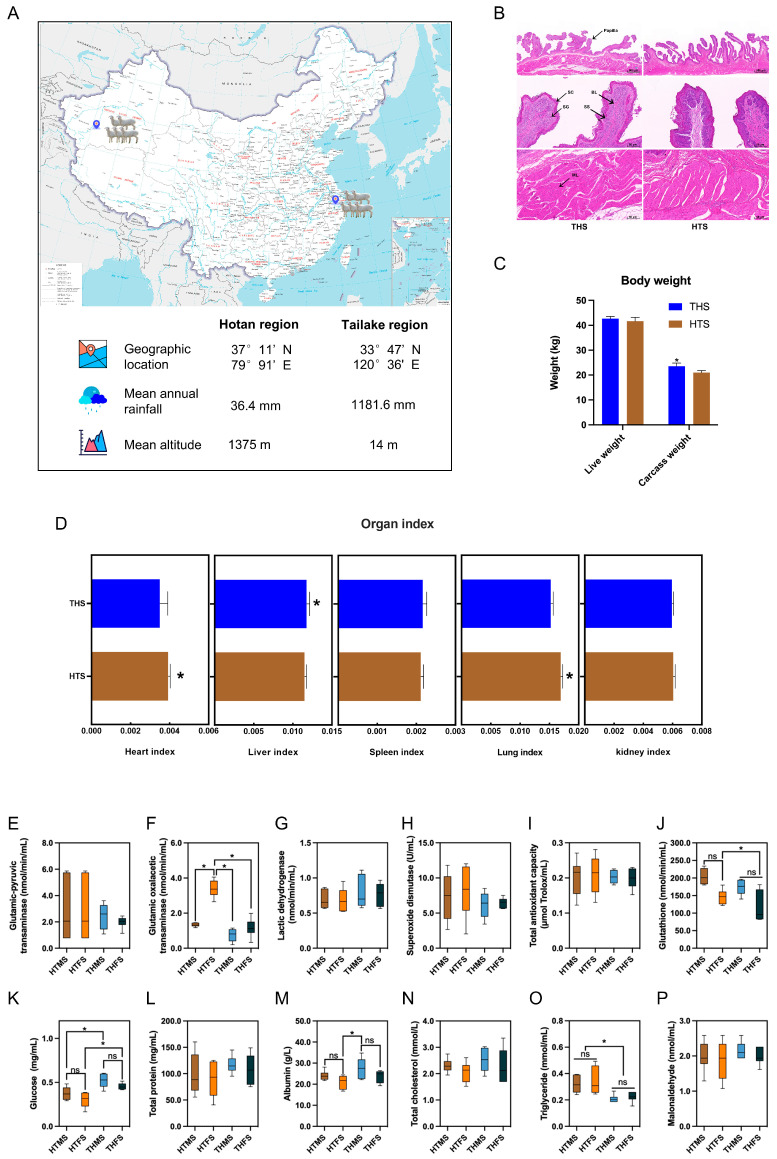
Comparison of growth performance, rumen tissue sections and serum biochemical indices of Hu sheep in the Taihu region (THS) and Hotan region (HTS). (**A**) Sample distribution diagram. (**B**) Rumen slices from Hu sheep from different regions, SC is the stratum corneum, SG is the stratum granulosum, SS is the stratum spinosum, BL is the basal layer, and ML is the muscle layer. (**C**) Live and carcass weights of Hu sheep in different regions. (**D**) Organ index of Hu sheep in different regions. (**E**–**P**) Serum biochemical indices of Hu sheep in different regions; HTMS is rams in the Hotan region, HTFS is ewes in the Hotan region, THMS is rams in the Taihu region, and THFS is ewes in the Taihu region. ns *p* > 0.1, * *p* < 0.05.

The rumen epithelial tissue structure of Hu sheep from two regions was different (Table S1, Figure 1B). The rumen basal layer (BL) and the stratum corneum (SC) of the Taihu region (THS) group was significantly higher (*p* < 0.05) than for the Hotan region (HTS), but the muscle layer (ML) (*p* < 0.01) and length of the papilla (LP) (*p* < 0.05) were significantly lower than for HTS, and there was no statistical difference in stratum granulosum (SG), stratum spinosum (SS) and width of the papilla (WP).

The carcass weight (*p* < 0.05, Figure 1C) and liver organ index (*p* < 0.05, Figure 1D) of Hu sheep in THS were significantly higher than those of Hu sheep in HTS. The heart and lung organ index of HTS were significantly higher than those of THS (*p* < 0.05, Figure 1D). There was no significant difference in the live weight, spleen and kidney organ index between the two groups (*p* > 0.05, Figure 1C,D).

Serum parameters for the different region groups are presented in Figure 1E–P. There were no significant differences in serum metabolic indices between different sexes of sheep in the same region, except for glutamic oxalacetic transaminase (Figure 1F). The glucose concentration of THS were significantly higher than those of HTS (*p* < 0.05, Figure 1K). The triglyceride concentration of HTS were significantly higher than those of THS (*p* < 0.05, Figure 1O). There was no significant difference in the other serum parameters between the two groups (*p* > 0.05, Figure 1E,G–I,L,N,P).

### 2.2. Genome Profiling of Rumen Microorganisms

**Figure 2:**
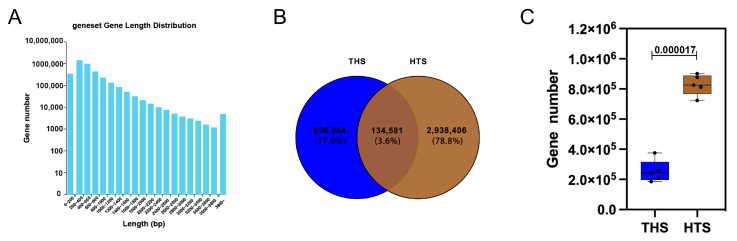
Metagenomic sequencing results. (**A**) Non-redundant gene length distribution map. (**B**) Venn map of gene number differences between groups. (**C**) Box map of gene number differences between groups.

After excluding low quality and n-containing reads, the metagenomic analysis of rumen contents in THS and HTS showed an average of 7,165,148,598 and 7,537,783,939 clean bases, respectively. Furthermore, the final effective reads obtained for subsequent analysis after removal of the host genome sequence were 46,941,261 and 49,711,564 for THS and HTS, respectively. Q20 and Q30 were >98% and >95%, respectively. (Table S2) These results indicate that the sequencing data are credible and can be used for subsequent bioinformatics investigation. The genome assembly of the Hu sheep in the Taihu region had a higher richness in diversity, with an average of 94.68% across samples, while the genome assembly of the Hu sheep introduced into the Hotan region had a lower richness in diversity, with an average of 72.27% across samples (Table S3), suggesting that the rumen macrogenome has undergone a major change after the ex situ survival of the Hu sheep. For THS and HTS, the predicted number of genes was 270,326 and 981,127, respectively (Table S4). The similarity threshold was 95%, and the coverage threshold was 90% (Figure 2A). The total number of genes in the non-redundant gene set was 3,729,051. According to the Venn, a total number of 134,581 genes were shared between THS and HTS, while 656,064 and 2,938,406 genes were unique to THS and HTS, respectively (Figure 2B). Obviously, the HTS group is more diverse (*p* < 0.001, Figure 2C).

### 2.3. Differences in Rumen Microbial Taxonomy

**Figure 3:**
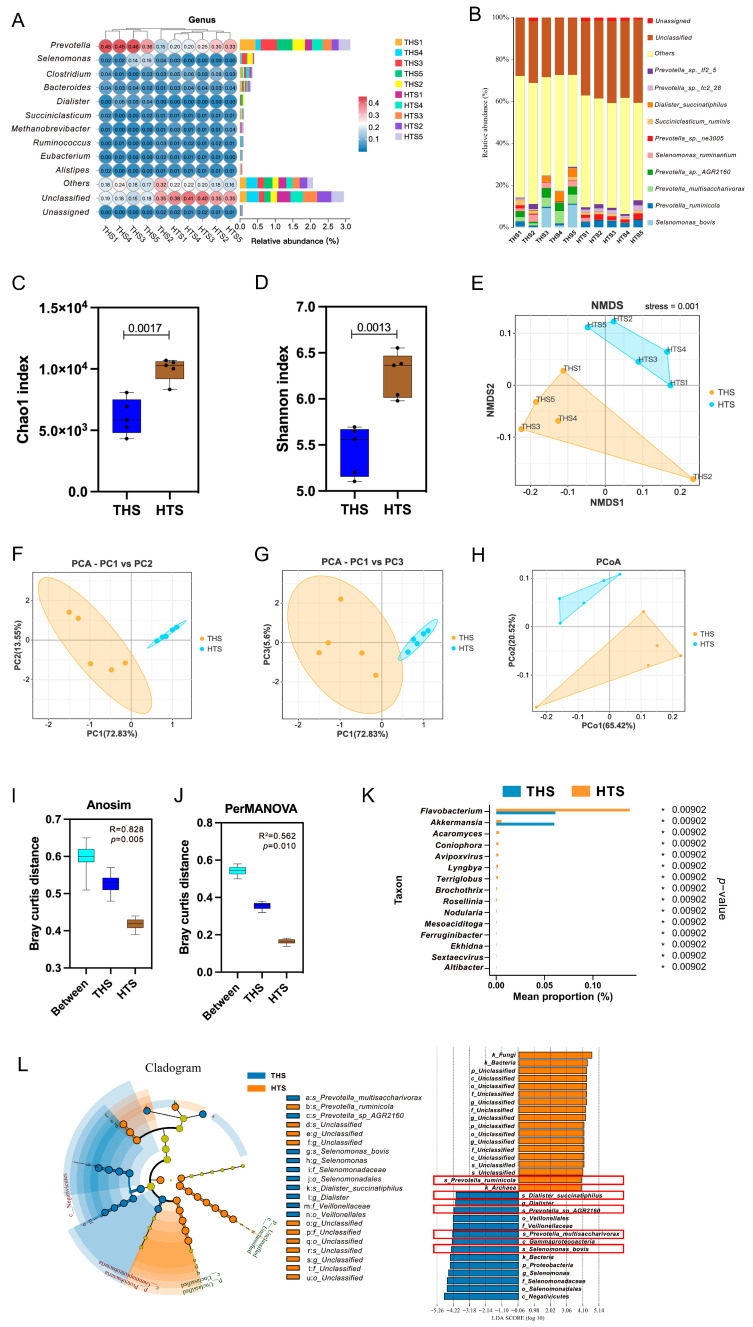
Analysis of microbial species composition in rumen. (**A**) Genus-level species composition and clustering analysis. (**B**) Species-level species composition. (**C**) Chao1 test. (**D**) Shannon test. (**E**) NMDS test. (**F**,**G**) PCA test. (**H**) PCoA test. (**I**,**J**) The analysis of intergroup disparities. (**K**) The top 15 levels exhibited significant disparities. (**L**) Line discriminant analysis of species level difference. * *p* < 0.05.

The non-redundant genes were compared with the species information of the sequence in the Non-Redundant Protein Database (NR, ftp://ftp.ncbi.nih.gov/blast/db (accessed on 13 July 2023)), and information on the species composition and relative abundance of the samples was obtained, as shown in (Table S5). The dominant bacterial phyla in the rumen were Bacteroidetes (THS: 48.53%, HTS: 47.92%) and Firmicutes (THS: 32.90%, HTS: 22.14%); the content of other phyla differed between the two groups. THS was followed by Proteobacteria (6.75%); HTS was followed by Unclassified (11.70%) and Chytridiomycota (2.72%). (Tables S6 and S7). The dominant bacterial generea were *Prevotella*, *Selenomonas*, *Clostridium* and *Bacteroides*. In addition, the cluster analysis shows good consistency, except for the THS2 samples (Figure 3A). At the species level, the dominant bacteria in the THS was *Selenomonas_bovis* (4.35%), and for HTS, it was *Prevotella_ruminicola* (2.94%), there is a clear difference in the species level between the two groups (Tables S8 and S9, Figure 3B).

Alpha diversity reflects the species richness and diversity of individual samples. The Chao1 index (*p* = 0.0017, Figure 3C) and Shannon index (*p* = 0.0013, Figure 3D) were significantly lower in the THS than in the HTS, indicating that the rumen of Hu sheep in the Hotan region had a relatively rich microbiota. Based on the Barry–Curtis distance, beta diversity analysis showed that non-metric multidimensional scaling (NMDS, stress = 0.001, Figure 3E), principal components analysis (PCA, Figure 3F,G) and principal co-ordinates analysis (PCoA, Figure 3H) reflected that the rumen microbial composition of Hu sheep in the same region was relatively similar, and sample aggregation was relatively high. On the other hand, the Anosim (*p* = 0.005, Figure 3I) and PerMANOVA (*p* = 0.01, Figure 3J) analyses showed that there were significant differences in the rumen microbiota of Hu sheep from different regions. All the evidence showed that the rumen microbiota of Hu sheep changed significantly to adapt to the high altitude and arid environment after survival in the Hotan region.

To find the differences in rumen microbial species of Hu sheep in different regions, the rank sum test was used for analysis, and the *p*-value was corrected. As can be seen from the result (Figure S1), samples from different regions are closely clustered and have obvious regional characteristics. After ranking in descending order according to the *p* value, the top 15 genera (*Flavobacterium*, *Akkermansia*, *Acaromyces*, *Coniophora*, *Avipoxvirus*, *Lyngbya*, *Terriglobus*, *Brochothrix*, *Rosellinia*, *Nodularia*, *Mesoaciditoga*, *Ferruginibacter*, *Ekhidna*, *Sextaecvirus* and *Altibacter*) were obtained as representatives to distinguish the rumen microorganisms of Hu sheep from the two regions (Table S10, Figure 3K). To determine the microbiota biomarkers adapted to the high altitude and arid environment of the Hu sheep, all microorganisms with statistical differences between the two groups were screened via line discriminant analysis (LDA) Effect Size (lefse) [28]. It was found that, except for unknown microorganisms, those with significantly higher HTS content than THS at the species level were *Prevotella_ruminicola*, and those with significantly higher THS content were *Dialister_succinatiphilus*, *Prevotella_sp__AGR2160*, *Prevotella_multisaccharivorax* and *Selenomonas_bovis* (Figure 3L). Therefore, the significant differences of these four microorganisms at the species level can be used as markers to distinguish the adaptability of Hu sheep.

### 2.4. Functional Maps and Functional Differences of the Rumen Microbiome

**Figure 4:**
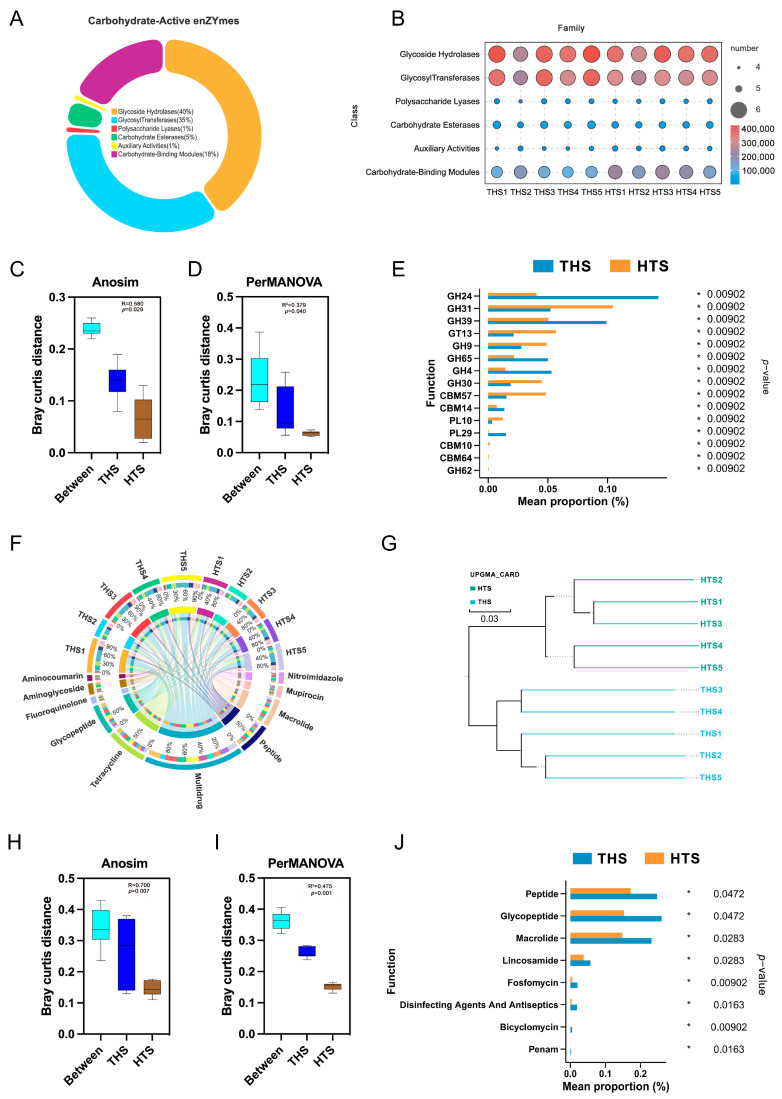
Differential analysis of rumen microbial function in sheep from different regions. (**A**) Compositional analysis of rumen microbial enzymes. (**B**) Analysis of enzyme components. (**C**,**D**) The analysis of intergroup enzyme disparities between Anosim and PerMANOVA. (**E**) Microbial enzymes analyzed in the top 15 differences. (**F**) Compositional analysis of rumen microbial CARD. (**G**) Cluster analysis of the CARD map. (**H**,**I**) The analysis of intergroup CARD disparities between Anosim and PerMANOVA. (**J**) The representative of differential antibiotic resistance. * *p* < 0.05.

The function of the rumen microbiome was determined by genes encoding Carbohydrate Active Enzymes (CAZyme) and the Comprehensive Antibiotic Research Database (CARD). For CAZyme analysis, the protein sequences of the non-redundant genes were annotated to six carbohydrate enzyme classes, accounting for 40% in Glycoside Hydrolases (GH), 35% in Glycosyl Transferases (GT) and 18% in Carbohydrate-Binding Modules (CBM) (Figure 4A), with smaller percentages in the other classes (Figure 4B). Anosim (*p* = 0.029, Figure 4C) and PerMANOVA (*p* = 0.040, Figure 4D) analyses of the annotated differential carbohydrate enzymes revealed significant differences in the rumen microbial carbohydrate function of lamb from different regions. According to rank sum test analysis, the carbohydrate enzymes with higher THS significance than HTS were GH24, GH39, GH65, GH4, CBM14 and PL29, while those with higher HTS significance were GH31, GT13, GH9, GH30, CBM57 and PL10 (Figure 4E). For CARD analysis, Peptide, Tetracycline and Macrolide were the most annotated resistances in both groups, and THS annotated more complex types (Table S11, Figure 4F). UPGMA (Figure 4G), Anosim (*p* = 0.007, Figure 4H) and PerMANOVA (*p* = 0.001, Figure 4I) analysis showed that the antibiotic genes were consistent in Hu sheep from the same area and that there were significant regional differences. Following an analysis of the differences between antimicrobial resistance (AMR), the rank sum test demonstrated that the frequency of resistance of THS was significantly higher than that of HTS in a number of categories, including peptide, glycopeptide, macrolide, lincosamide, fosfomycin, disinfectants and antiseptics, bicyclomycin and penam (Figure 4J).

### 2.5. Rumen Metabolome Analysis

**Figure 5:**
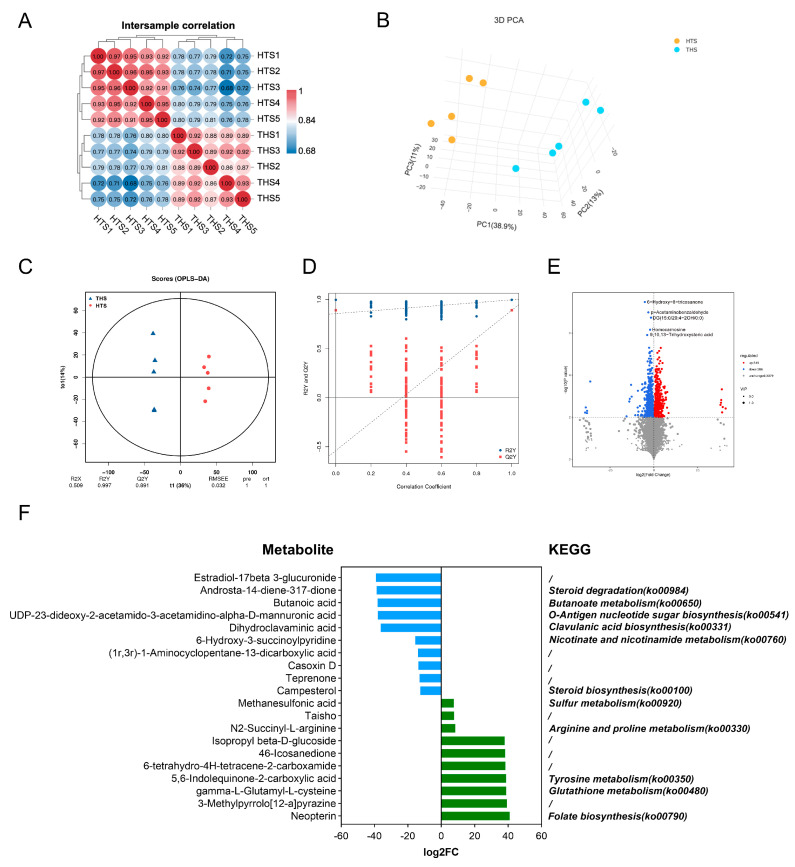

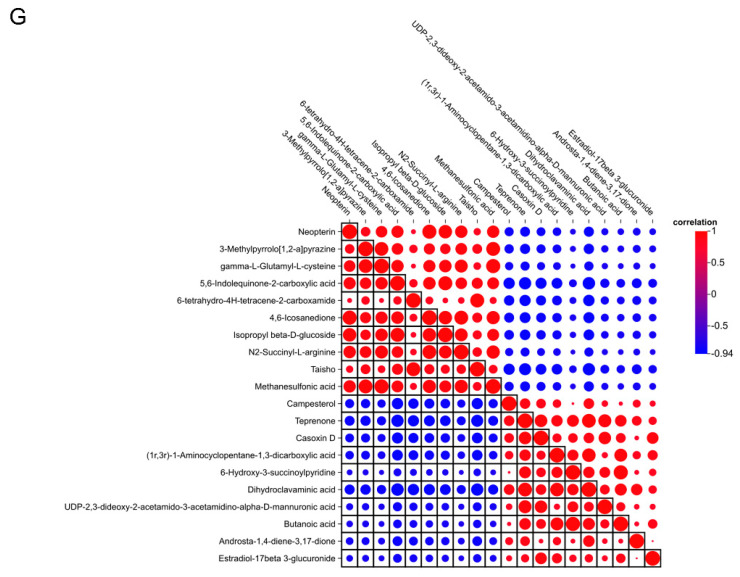
Microbial metabolomics analysis of the rumen. (**A**) Cluster analysis of the microbial metabolome in sheep rumen. (**B**) PCA test. (**C**,**D**) The analysis of intergroup disparities. (**E**) Volcanic map of differential rumen microbial metabolism of Hu sheep in two groups. (**F**) Differential metabolites upregulated 20 and downregulated 20 and predicted KEGG pathways. (**G**) Correlation analysis of differential metabolites between upregulated 20 and downregulated 20.

Based on the LC-QTOF platform, a total of 16,491 peaks were detected, of which 4111 were metabolites (Table S12). Spearman’s rank correlation coefficient (Figure 5A) and PCA (Figure 5B) showed that the samples in the two groups had good repeatability and that the rumen metabolite clustering of Hu sheep in the two regions had regional characteristics. The OPLS-DA [29] score map showed that both groups could separate rumen metabolites (Figure 5C,D). Based on the OPLS-DA model, metabolites with fold change (FC) ≥ 1 were selected for between-group difference analysis. A total of 732 differential metabolites were found, of which 346 were upregulated and 386 were downregulated (Figure 5E). According to the size of the FC, 10 upregulated and 10 downregulated metabolites were selected and annotated to 11 KEGG pathways (Figure 5F). Correlation analysis of these 20 differential metabolites showed that upregulated metabolites were generally negatively correlated with downregulated metabolites (Figure 5G). 4,6-Icosanedione, Methanesulfonic acid and N2-Succinyl-L-arginine, as representatives of upregulated metabolites, and campesterol, teprenone and dihydroclavaminic acid, as representatives of downregulated metabolites, were highly correlated with other metabolites (Table S13). These six metabolites could be used as markers of the adaptability of the Hu sheep rumen microbiome in arid and high-altitude environments.

### 2.6. Combined Metagenome and Metabolome Analysis

**Figure 6:**
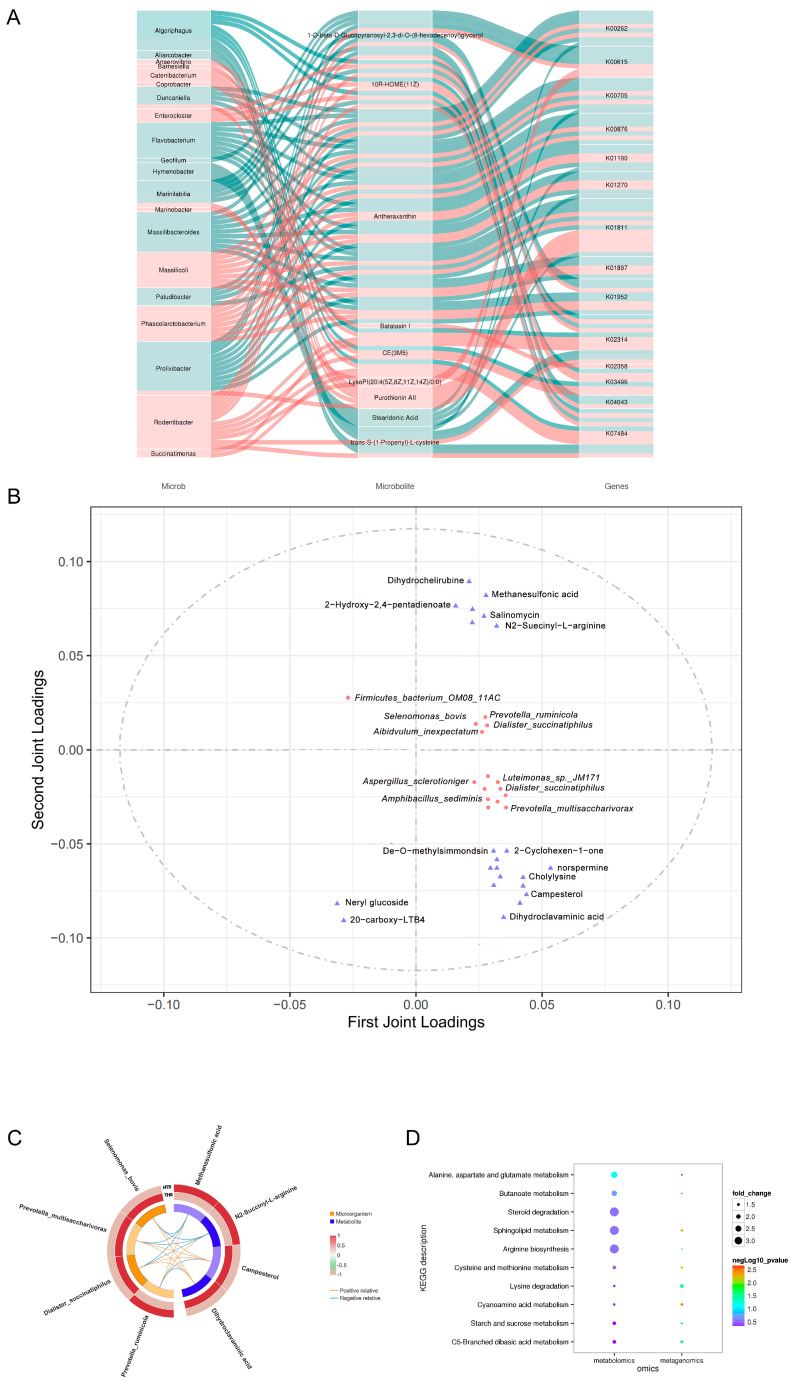
Combined metagenome and metabolome analysis. (**A**) Targeted relationships between microbial populations, metabolites and genes. (**B**) Metagenome and metabolome O2PLS analysis. (**C**) Correlation analysis between target metabolites and microorganisms. (**D**) Joint KEGG of metagenomics and metabolomics.

The data of the two omics were analyzed to examine whether there is a linkage effect between the two omics. Based on the correlation via SPSS (version 22.0), the relationship between metabolites and microbial strains and between metabolites and functional genes can be obtained, and the multi-level regulatory relationship between strains, functional genes and metabolites can be sorted out (|r| > 0.8, *p* < 0.05, Figure 6A). Metabolomic and metagenomic data analyzed via PCA are highly reliable (Figure S2). The O2PLS analysis revealed the top 25 microorganisms and metabolites with the largest linkage effect (Figure 6B). Combined with five species with significant differences in metagenomic screening and six metabolites with significant differences in metabolomic screening, O2PLS showed four species and four metabolites each, and after Pearson analysis, it was found that the rumen microbiota and metabolite content of HTS are significantly different from THS. On the other hand, *Prevtella_ruminicola* from HTS showed a strong positive correlation with methanesulphonic acid and N2-succinyl-L-arginine. There was a strong negative correlation with campesterol and dihydroclavaminic acid. Interestingly, the other three showed the exact opposite with *Prevtella_ruminicola* (Figure 6C). These four species and four metabolites are the key indicators of Hu sheep adaptation in the Hotan area. For KEGG analysis, steroid degradation, sphingolipid metabolism and arginine biosynthesis were obtained by annotating the co-significance of differential metabolites and differential species (Table S14, Figure 6D). Hu sheep may regulate rumen function through these pathways to survive in drought and at high altitude.

### 2.7. Correlation Analysis of Microorganisms, Metabolites and Phenotypes

**Figure 7:**
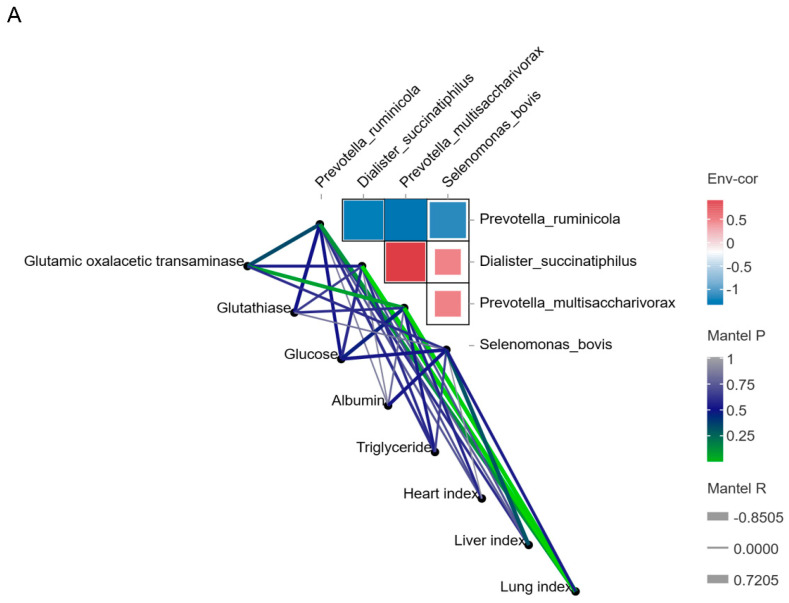

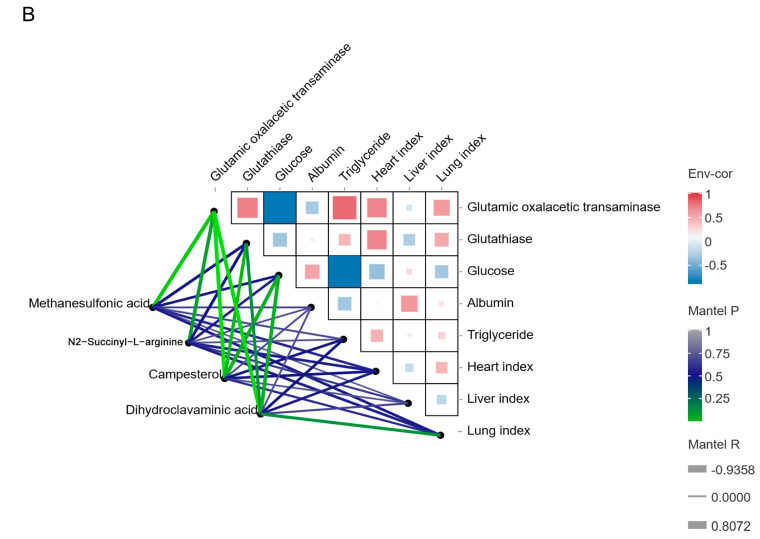
Correlation analysis between microorganisms, metabolites and phenotype. (**A**) Correlation analysis between microorganisms and phenotype. (**B**) Correlation analysis between metabolites and phenotype.

To understand the effects of the four screened metabolites and four microorganisms on the adaptability of Hu sheep to drought and high-altitude environments, a correlation analysis was performed. The upregulated species *Prevotella_ ruminicola* in the HTS group was significantly negatively correlated (r ≤ 0.6) with the other three species. *Prevotella_ruminicola* was positively correlated with glutamic oxalacetic transaminase (r ≥ 0.6, *p* < 0.05), glutathiase (r ≥ 0.6, *p* > 0.05), triglyceride, the heart index and the lung index. It was negatively correlated with glucose, albumin and the liver index. The other three downregulated species were positively correlated with glucose and albumin and negatively correlated with glutamic oxalacetic transaminase and glutathiase (Figure 7A). More interestingly, the correlation between the upregulated metabolites methanesulfonic acid and N2-succinyl-L-arginine with serum biochemical indices and organ indices was opposite to that of the downregulated metabolites campesterol and dihydroclavaminic acid (Figure 7B). Glutamic oxalacetic transaminase was the most highly correlated (|r| ≥ 0.7, *p* < 0.05) with these four metabolites, followed by glutathiase (|r| ≥ 0.5, *p* > 0.05) and glucose (|r| ≥ 0.5, *p* > 0.05).

## 3. Discussion

In this study, metagenomic sequencing and metabolome sequencing technologies were combined to investigate the environmental adaptability of rumen microorganisms in Hu sheep from different regions, which provided a good angle to explore sheep quality traits and a way to explore the adaptive evolution of ruminants.

The Hu sheep is a world-renowned breed known for its high fertility and is a unique sheep genetic resource in China. It has been recognized as one of the first national livestock and poultry genetic resource conservation species. Originating from the agricultural area of South China, the Hu sheep is a local breed developed through long-term indoor rearing and artificial breeding. Its name is derived from the fact that it is mainly raised in the Taihu region of Jiangsu, Zhejiang and Shanghai [30,31]. Hu sheep are suitable for year-round indoor feeding, can adapt to the high-temperature and high-humidity environment in the south, and have strong stress resistance. Early growth and development is rapid; 6-month-old lambs can reach more than 70% of the annual sheep weight, adult male and female sheep can reach 65 kg and 45 kg, respectively. The Hu sheep is known for its docile temperament, early sexual maturity and strong maternal instincts. Both male and female ewes reach sexual maturity at 5–6 months of age and exhibit perpetual estrus, resulting in a high number of lambs per litter with an average lambing rate of up to 230% [32,33]. Hu sheep have been successfully introduced into different regions of China and have shown excellent adaptation to the local environment. However, there is a lack of research on the environmental adaptation of Hu sheep, particularly about the ruminant rumen microbial population. Natural selection of some environmentally adapted breeds has led to the identification of important traits, both morphological and physiological, that may promote genotype × environment interaction. The ability of an animal to adapt to its environment is first manifested in its phenotypic traits, including body size [34], coat [35] and respiratory [36] and digestive [37,38] systems. Generally, it takes about three generations for this adaptation to continue and evolve [39,40]. Therefore, we selected male lambs from the Hotan region (HTS) that had lived for more than three generations and compared them with male lambs from the original Taihu region (THS). In our study, no significant difference in live weight was found after the Hu sheep in the two regions were managed with the same feeding methods. However, in terms of carcass weight, the Hu sheep from the source area were still large, and the organ index of the liver was also higher than that of the Hu sheep from the Hotan area. All kinds of evidence showed that the meat production performance of the Hu sheep in the Hotan area decreased after the introduction of the Hu sheep in the Hotan area. This may be due to changes in metabolic patterns. To this end, we observed the rumen slices of the Hu sheep in two regions, and the results showed that to adapt to the arid nutritional environment, the rumen papilla length and muscle layer of the Hu sheep were higher than those of the original sheep. In Liu’s study [41], hay stimulated changes in the width and height of the rumen papillas of Tibetan sheep. All the evidence suggested that the rumen tissues of the Hu sheep had been altered and, accordingly, the environment of the rumen microorganisms could be driven by the environment to adapt. Through our biochemical analysis of the serum of Hu sheep in the two regions, we found that the serum glucose content of Hu sheep in the Hotan region was lower than that of the place of origin, indicating that the body metabolism of Hu sheep was accelerated when they were introduced to arid and high altitude areas, and the triglyceride concentration was increased, strongly suggesting that fat deposition was higher than that of the place of origin, which may be the reason for the reduced meat production performance.

To investigate the energy metabolism and adaptability of the Hu sheep to the foraging environment, we mainly studied the ruminant rumen microbial population. According to the Venn, a total of 134,581 rumen microbial genes were shared between THS and HTS, while 656,064 and 2,938,406 genes were unique to THS and HTS, respectively. Significant changes in the rumen microbiota occurred after sheep were moved to different locations where the environment changed significantly. Similar to Zhong’s report [42], environmental changes in temperature and humidity lead to heat stress-induced adaptation of rumen microbes in goats. As in many previous studies that have investigated rumen microbiomes using metagenomics [43], bacteria were the most abundant rumen microbial kingdom in the sheep rumen, and the differences in rumen microbial characteristics between THS and HTS were mainly found in bacteria. At the kingdom level, the rumen microbial communities of sheep in the two regions exhibited notable differences, with the discrepancies becoming more pronounced at the genus and species levels (Table S5). Bacteroidetes and Firmicutes were dominant in both regions. Many studies [44,45] have shown that Bacteroidetes and Firmicutes drive the rumen microenvironment with different microbial diversity and community composition. The dominant bacterial genera in the rumen were Prevotella, Selenomonas, Clostridium and Bacteroides. At the species level, the dominant bacteria in THS was Selenomonas_bovis (4.35%) and in HTS was Prevotella_ruminicola (2.94%); there is a clear difference at species level between the two groups. Chai’s study [24] showed that Prevotella_ruminicola in the goat rumen was strongly correlated with cellulose degradation after feeding. Combined with our research results, the rumen microbiota of Hu sheep changed significantly to adapt to the high altitude and arid environment after survival in the Hotan region. To find significant differences in the rumen microbiota between the two regions, 15 genera and five species were identified via rank sum test.

Carbohydrate Active Enzymes (CAZymes) are essential for microbial communities to thrive in carbohydrate-rich environments such as animal guts, agricultural soils, forest soils and marine sediments [46]. CAZymes are a class of enzymes that break down the glycosidic bonds between polysaccharides. The complexity of the polysaccharide structure determines the diversity of CAZymes required for degradation. Currently, CAZymes can be divided into six categories: glycoside hydrolases (GHs), glycosyltransferases (GTs), polysaccharide lyases (PLs) and carbohydrate esterases (CEs), carbohydrate-binding modules (CBMs) and auxiliary REDOX reductases (AAs) [47]. In this study, the function of the rumen microbiota in both regions was annotated in the same category, indicating that the function of the rumen microbiota was relatively stable and mainly concentrated in GHs, GTs and CBMs. GHs can break glycosidic linkages via hydrolysis [48,49]. The results of our study demonstrated a notable increase in rumen carbohydrate enzymes in GH31 and a pronounced decline in GH24 and GH39 in Hu sheep inhabiting arid regions. These results indicate that the rumen of Hu sheep has undergone significant changes in response to forage scarcity and carbohydrate degradation due to long-term drought stress, thereby adapting to selection pressure. GTs catalyze the activation of sugar moieties, attaching them to specific receptor molecules to form glycosidic bonds, and play a crucial role in carbohydrate synthesis, as well as in the adaptability and pathogenicity of host microorganisms [50]. It is worth noting that in our study, GT13 was significantly upregulated in the rumen of Hu sheep in arid areas, which may play an important role in carbohydrate synthesis such as grape, which may serve as an enzymatic marker to distinguish whether an organism can survive in arid areas. CEs primarily remove polysaccharide ester groups and participate in the degradation of carbohydrate side chains, promoting the cleavage of glycosidic bonds by GHs and PLs [51]. PLs can disrupt glycosidic bonds through a complex mechanism [52]. CBMs have no catalytic activity themselves, but assist GHs, PLs and other hydrolytic enzymes by anchoring CAZymes to the substrate surface, increasing contact time and surface area [53,54]. CBM57 (upregulated in arid regions) and CBM14 (downregulated in arid regions) play important roles in the response of sheep to drought stress. AAs can participate in the modification of lignin, thus breaking the barrier of plant biomass resistance to degradation and accelerating substrate hydrolysis [55]. Therefore, changes in rumen microorganisms lead to changes in microbial carbohydrate enzymes, thus promoting the degradation of feed by sheep under drought stress, especially the hydrolysis and synthesis of carbohydrate substances, accelerating energy metabolism and promoting fat deposition in sheep under harsh environments, thus affecting the growth performance and production performance of sheep.

For the Comprehensive Antibiotic Research Database (CARD) analysis, peptide, tetracycline and macrolide resistances were the most annotated in both groups, and THS annotated more complex types. Antibiotics refer to a class of secondary metabolites produced by microbial life processes, a class of chemical substances that can inhibit the growth or activity of other microorganisms or even kill other microorganisms [56]. Antimicrobial resistance (AMR) is a naturally occurring phenomenon that was widespread in the environment before the selective pressure of antibiotic use, but the irrational use of antibiotics accelerates the emergence and spread of bacterial resistance. In the arms race between antibiotics and bacteria, humans are rarely ahead [57]. The irrational use of antibiotics promotes the natural evolution of bacteria, helping microbes to develop resistance to these infection-fighting drugs, and the rate at which bacteria develop resistance has outpaced the development of new antibiotics. Antimicrobial resistance in bacteria is mainly achieved through antibiotic resistance genes (ARGs). Drug resistance in pathogenic bacteria is a growing threat to global health, and the development and spread of drug resistance in microorganisms is largely due to the misuse and abuse of antibiotics [58]. Due to the different environment in different regions, there are large differences in antibiotic use patterns on farms, so that AMR in animals has a regional specificity. In this study, antibiotic resistance was different in different regions of sheep and could be grouped in the same region, suggesting that rumen microorganisms had changes in resistance to antibiotic pressure different from the place of origin in the face of the health risk of drought environmental stress. Studies have shown that rumen microorganisms in yaks on the Qinghai-Tibet Plateau have evolved resistance to tetracycline in response to the threat of exogenous antibiotics [59,60]. In this study, eight AMRs in the rumen microbiota of Hu sheep in arid areas showed significant changes with respect to origin, among which the levels of peptides, glycopeptides and macrolides were more abundant. Antimicrobial peptides (AMPs) are molecules with broad-spectrum activity against bacteria, fungi, protozoa and viruses. AMPs are essential mechanisms of the innate immune response; these molecules are found at strategic sites in animals, mainly on epithelial surfaces. The best characterized AMPs were the sheep myeloid AMPs (SMAP-29 and SMAP34) because of their broad-spectrum antimicrobial activity against gram-positive and gram-negative bacteria and fungi [61]. Given the broad-spectrum nature of peptide antibiotics, the emergence of resistance is a significant indicator of changes in sheep adaptation to environmental changes. Glycopeptides, a class of cell wall biosynthesis inhibitors, have been the antibiotics of choice against drug-resistant gram-positive bacterial infections. Recent evidence shows that some glycopeptides have antimicrobial activity against gram-negative bacteria, mycobacteria and viruses, extending their spectrum of activity across the microbial kingdom [62]. The immunomodulatory effects of macrolide antibiotics in respiratory disease have been widely reported, particularly since COVID-19 [63]. This evidence suggests that environmental stress in sheep is forcing changes in microbial vitamin resistance to adapt to the arid environment. It also suggests that we can further explore high quality antibiotic resistance genes in sheep.

In this study, a total of 732 differential metabolites were found, of which 346 were upregulated and 386 were downregulated. After significance analysis, we focused on six metabolites, and when combined with metagenomic analysis, we found that four metabolites (methanesulfonic acid, N2-succinyl-L-arginine, campesterol and dihydroclavaminic acid) could be better correlated with four microbial species (*Prevotella_ruminicola*, *Dialister_succinatiphilus*, *Prevotella_multisaccharivorax* and *Selenomonas_bovis)*. Methanesulfonic acid is closely related to the formation of methane, and ruminants are one of the largest sources of global CH_4_ emissions. This enteric CH_4_ is produced exclusively by methanogenic archaea as a natural product during microbial fermentation in the reticulorumen [64]. The formation of CH_4_ results in a gross energy loss to the ruminant host and is also an environmental concern. The present study showed that the pathway involved in methanesulphonic acid is Sulphur metabolism [65], suggesting that the formation of methane may be related to the metabolism of hydrogen, carbon dioxide and simple organic substances (such as methanol, methylamine and dimethyl Sulphur) in the body. Interestingly, in this study, methanesulphonic acid was correlated with glutamic oxalacetic transaminase activity, and both were upregulated in the rumen of Hu sheep in arid areas. Glutamic oxalacetic transaminase is an important index of organic matter, protein and fat conversion in livestock and poultry and is closely related to heart and liver function [66]. In our study, the increased activity of glutamic oxalacetic transaminase in the serum of Hu sheep introduced to the Hotan area may indicate that the liver of Hu sheep in the Hotan area is in a state of high activity. In particular, the liver index of sheep in arid areas decreased, and the liver may be under stress at this time, and energy metabolism is accelerated under harsh conditions, which may be the cause of fat deposition in sheep in high altitude and arid areas. In addition, our study showed that there was also a strong correlation between glutamic oxalacetic transaminase activity and *Prevotella_ruminicola*. The high abundance of *Prevotella_ruminicola* in the rumen microorganisms of sheep in arid areas means that this bacterium can be used as a biomarker of rumen microorganisms in ruminants in arid areas. Currently, the sheep’s body metabolism is accelerated in response to the environment, which also accelerates the activities of the sheep’s rumen methane bacterial community. Although metabolism is accelerated, it is not fully metabolized and more methane is produced, which is harmful to the environment. Finding ways to reduce the role of *Prevotella_ruminicola* in ruminant diets in arid areas could reduce the methane threat.

In addition to the accelerated metabolism of sheep in arid areas, another change is an increase in antioxidant capacity. In this study, *Prevotella ruminicola* as a marker bacterium was also strongly correlated with glutathione peroxidase activity. Studies have shown that drought signals stimulate the body’s antioxidant system to maintain REDOX homeostasis and prevent cell damage through glutathione peroxidase to cope with the challenges of extreme environments [67,68]. Meanwhile, as an essential amino acid for optimal growth and nitrogen balance in young mammals, arginine plays an obvious role in promoting growth, improving immunity and fertility and enhancing antioxidant capacity [69]. There are many metabolic pathways of arginine in the body that can produce creatine, polyamines, nitric oxide and other important functional substances that can improve nitrogen utilization and reduce nitrogen excretion [70,71]. In this study, KEGG analysis of metagenomics and metabolomics differences between the two regions pointed to the arginine biosynthesis pathway, and our results showed that it was mainly reflected in the succinylation of arginine, which greatly accelerated ammonia metabolism in sheep from arid regions, which may also be one of the reasons why the rumen produced more methane after sheep from different places were introduced. At the same time, arginine succinylation has a strong relationship with glutamic oxalacetic transaminase activity. It also has a strong correlation with methanesulphonic acid, which may be an auxiliary effect with the metabolism of methanesulphonic acid. In addition, the abundance of N2-succinyl-L-arginine and glutathione activities increased in the rumen of sheep from arid areas, suggesting that the antioxidant capacity of sheep was significantly improved to adapt to the dry and high-altitude environment.

Some studies [72] had shown that campesterol in the intestinal tract of animals has a strong correlation with immunity, which is inversely proportional to *Escherichia coli*, and campesterol has also been reported [73] to be related to the protective barrier of the intestinal tract of sheep fetuses. Campesterol is negatively correlated with *Dialister_succinatiphilus*, *Prevotella_multisaccharivorax* and *Selenomonas_bovis*, and in our study, there is a strong inverse correlation with triglyceride, suggesting that campesterol has an inverse effect on animal lipid metabolism. Campesterol in the rumen of sheep in arid areas is lower than in the place of origin, suggesting that the reduction in campesterol promotes fat deposition in sheep to cope with drought stress. Dihydroclavaminic acid (DHCA) is commonly used as an intermediate in the biosynthesis of beta-lactam antibiotics. These include common antibiotics such as penicillin and cephalosporins. DHCA acts as an important intermediate in the biosynthetic pathway of these antibiotics by participating in the catalytic action of several enzymes that ultimately transform into mature antibiotic structures [74]. In addition to its role in antibiotic biosynthesis, some studies have suggested that DHCA may have a regulatory role in apoptosis and cell proliferation [75]. However, further studies are needed to clarify the specific function and mechanism of DHCA in animals, particularly its role in the metabolic and physiological processes of intestinal microorganisms. In this study, DHCA is positively correlated with *Dialister_succinatiphilus*, *Prevotella_multisaccharivorax* and *Selenomonas_bovis.* It is interesting to note that DHCA is directly proportional to the lung index, suggesting that DHCA may be related to adaptation of the lung environment in sheep and may also have a strong relationship with resistance.

Another manifestation of the harsh environmental stress experienced by arid zone sheep may be changes in nerve conduction. In this study, metagenomic and metabolomic differences in sheep microbes from two regions pointed to both steroid degradation and sphingolipid metabolic pathways. Studies have shown that there is a relationship between steroid degradation pathways and nerve conduction, particularly during regulatory processes in the nervous system. For example, when cortisol reaches a certain concentration in the nervous system, it affects the excitability and inhibition of neurons [76], and changes in the activity of some enzymes, particularly oxidase, may affect the rate of steroid metabolism and degradation and then the levels of steroids in the nervous system [77]. This may affect nerve conduction. There is a close relationship between the sphingolipid pathway and nerve conduction. Sphingolipid synthesis is a complex process involving many enzymes and metabolic pathways, including fatty acid synthesis, phospholipid synthesis, cholesterol synthesis, etc. Sphingolipid synthesis directly affects the formation and function of neuronal myelin. Our study provides evidence for adaptive changes in the animal nervous system in response to drought stress.

## 4. Materials and Methods

All procedures used in this study were approved by the Animal Care and Use Committee of Xinjiang Agricultural University (Approval number: 2024010).

### 4.1. Animals

The test animals were taken from Hu sheep farms in different parts of China, one in the Hotan region of Xinjiang (37°11′ N, 79°91′ E; mean annual rainfall, 36.4 mm; mean altitude, 1375 m) and the other in the Taihu region of Jiangsu (33°47′ N, 120°36′ E; mean annual rainfall, 1181.6 mm; mean altitude, 14 m) (Figure 1A). Sixty newborn healthy Hu lambs (30 males and 30 females) were weaned from each farm and fed the same diet until 8 months of age. The lambs are housed with the ewes and breastfed after birth, supplemented with the same starter feed from 15 days of age and weaned uniformly at 60 days of age. The main nutrients are listed in Table S15. Feed refusals were measured daily before morning feeding. Each daily ration was approximately 3% of body weight, with the amount offered adjusted daily to provide a slight surplus each day. Sheep were fed twice a day, at 6:00 am and 6:00 pm. The sheep were allowed to always eat and drink freely. All sheep were healthy and were not administered probiotics or antibiotics.

### 4.2. Sample Collection and Processing

At 3, 5 and 7 months of age, 10 mL of blood was collected from the cervical vein of Hu sheep after 24 h of fasting and divided into two parts, one-part anticoagulant whole blood and one-part procoagulant isolated serum, and stored at −20 °C. At 8 months of age, five rams were randomly selected from different areas and weighed, then ruminal fluid was collected deep in the mouth using a sheep ruminal sampling tube and quickly placed in liquid nitrogen. Upon return to the laboratory, they were transferred to the −80 °C ultra-low-temperature refrigerator for subsequent metagenomic and metabolomic analysis. After slaughter through the carotid artery, the weight of the heart, liver, spleen, lung and kidney was weighed. The organ index was calculated according to the following formula: individual organ weight/live weight. Rumen tissues were collected at the same sites for sectioning. The same farm was selected for each region for the purposes of this study.

### 4.3. Micromorphological Structure of the Rumen

Tissue from the rumen was cut into 1 cm × 1 cm tissue blocks and fixed in 4% paraformaldehyde. After fixation for 24 h, the sections were dehydrated, made transparent, waxed, embedded, sectioned and stained with hematoxylin and eosin to produce HE sections; hematoxylin stains the nucleus blue-violet, and eosin stains the cytoplasm pink. Rumen muscularis thickness, papillary height, papillary width, stratum corneum thickness, stratum granulosum thickness, stratum spinosum thickness and basal layer thickness were measured using the CaseViewer slice analysis system [78].

### 4.4. Biochemical Index Determination

Serum concentrations of 12 biochemical indices (glutamic-pyruvic transaminase, glutamic-oxalacetic transaminase, lactic dehydrogenase, superoxide dismutase, total antioxidant capacity, glutathione, glucose, total protein, albumin, total cholesterol, triglyceride and malonaldehyde) were determined via competitive enzyme-linked immunosorbent assay (ELISA) (Ruixin, Quanzhou, China) according to the manufacturer’s instructions.

### 4.5. DNA Extraction, High-Throughput Sequencing and Bioinformatics

The microbial genome was extracted using the TIANamp Genomic DNA DP705 kit (TIANGEN Bio Tek Co., Ltd., Beijing, China) in accordance with the manufacturer’s instructions. The DNA was quantified via UV spectrophotometry (Thermo Fisher Scientific Inc., Waltham, MA, USA). The sequencing library was prepared using the VAHTS Universal Plus DNA Library Prep Kit (ND617-02). The sequencing was performed on a MiSeq sequencer (Illumina BioTek Inc., San Diego, CA, USA) using the 2 × 300 bp paired-end sequencing strategy.

Utilize the fastp software (v0.23.4) [79] for filtering raw tags to obtain high-quality clean tags. Employ bowtie2 [80] to compare with the sheep genome (*ARS-UI_Ramb_v3.0*) sequence for host contamination removal, ensuring comprehensive quality control, noise reduction, splicing and chimera generation. Macrogenome assembly was performed using MEGAHIT software (v1.2.9) [81] to filter out contig sequences shorter than 300 bp. The results of the assembly were evaluated using QUAST software (v5.2.0) [82]. MetaGeneMark software (http://exon.gatech.edu/meta_gmhmmp.cgi (accessed on 6 October 2023), version 3.26) was used to identify coding regions in the genome using default parameters. Redundancy was removed using MMseqs2 software (https://github.com/soedinglab/mmseqs2 (accessed on 17 October 2023), v12-113e3). Representative sequences of the non-redundant gene catalogue were aligned with the NCBI NR database using BLASTP (version 2.2.28+) to obtain annotation results and species abundance degree. The e-value cut-off for the best match was set at 1 × 10^−5^. The microbial community was analyzed at multiple taxonomic levels, including kingdom, phylum, class, order, family, genus and species.

Alpha diversity metrics, including the Richness, Chao1 and Shannon index, were calculated based on the ASV table using the “diversity” function in the Vegan R package (https://rdrr.io/cran/vegan/, accessed on 24 October 2023). The principal coordinates analysis (PCoA) utilizing the Bray–Curtis dissimilarity matrix was calculated using the “pcoa” function in the ape R package (https://cran.r-project.org/web/packages/ape/index.html, accessed on 28 October 2023). We used the Bray–Curtis distance algorithm to perform PCA and cluster dendrogram analysis, using the unweighted pair-group method to calculate the arithmetic means. LEfSe was used to conduct simultaneous differential analysis of all classification levels of microorganisms. Linear discriminant analysis (LDA) > 2 was used as the screening criterion. We selected the Random Forests algorithm for analysis to obtain important microorganisms. Non-metric multidimensional scaling (NMDS) plots of the Bray–Curtis metric were calculated with square root transformed data in R (vegan package). A BLAST search (version 2.2.28+) with an optimization criterion cutoff of 1 × 10^−5^ was performed using USEARCH (http://www.drive5.com/usearch/, accessed on 30 October 2023) CAZY annotation and CARD (https://card.mcmaster.ca/, accessed on 30 October 2023).

### 4.6. Analysis of the Rumen Metabolome

The metabolome of the rumen was chromatographically separated using ultra-high performance liquid chromatography (UHPLC) and analyzed via mass spectrometry with a Waters Xevo G2-XS QT high-resolution mass spectrometer (Waters Acquity UPLC HSS T3). Waters Xevo G2-XS QTOF high resolution mass spectrometer can collect primary and secondary mass spectrometry data in MSe mode under the control of the acquisition software (MassLynx V4.2, Waters). In each data acquisition cycle, dual-channel data acquisition can be performed on both low collision energy and high collision energy at the same time. The raw data collected using MassLynx V4.2 is processed with Progenesis QI software (v3.0) for peak extraction, peak alignment and other data processing operations based on the Progenesis QI software (v3.0) online METLIN database and Biomark’s self-built library for identification, and at the same time, theoretical fragment identification and mass deviation all are within 100 ppm.

After normalizing the original peak area information with the total peak area, the follow-up analysis was performed. Principal component analysis and Spearman correlation analysis were used to judge the repeatability of the samples within group and the quality control samples. The identified compounds are searched for classification and pathway information in the database of the Kyoto Encyclopedia of Genes and Genomes (KEGG, http://www.genome.jp/kegg/, accessed on 15 November 2023), HMDB (https://hmdb.ca/, accessed on 17 November 2023) and lipid maps databases (https://lipidmaps.org/, accessed on 17 November 2023). According to the grouping information, calculate and compare the difference multiples; a *t*-test was used to calculate the difference significance p value of each compound. The R language package ropls (v1.35.4) was used to perform OPLS-DA modeling, and 200 permutation tests were performed to verify the reliability of the model. The VIP value of the model was calculated using multiple cross-validation. The method of combining the difference multiple, the *p* value and the VIP value of the OPLS-DA model was adopted to screen the differential metabolites. The screening criteria are FC > 1, *p* value < 0.05 and VIP > 1. The difference metabolites of KEGG pathway enrichment significance were calculated using the hypergeometric distribution test. The OmicShare Tools (https://www.omicshare.com/, accessed on 26 November 2023) were employed to perform the two-way orthogonal partial least squares (O2PLS) analysis.

### 4.7. Statistical Analysis

SPSS version 22.0 was used for independent variance *t*-test and correlation. Phenotypic data between pair of groups were analyzed and visualized using the multiple *t*-test method of GraphPad Prism (version 9.0, San Diego, CA, USA). *p* < 0.05 was considered significant for the analysis.

## 5. Conclusions

This study determined the serum biochemical characteristics of Hu sheep from different regions and the taxonomic characteristics, functions, metabolites and their interactions with metabolites of rumen microbes that contribute to the environmental adaptation of sheep. After introduction of Hu sheep to the relatively high-altitude, arid environment, changes in rumen tissues, metabolism and microbial populations have allowed the sheep to adapt quickly to the environment with improved growth performance. The main difference in the microbial population was *Prevotella_ruminicola*. In addition, methanesulphonic acid, N2-succinyl-L-arginine, campesterol and dihydroclavaminic acid were affected. The adaptive changes in the rumen microbial community of sheep face the stress of the drought environment by regulating the acceleration of body metabolism, the enhancement of antioxidant capacity and the alteration of nerve conduction. These results not only provide clues to the mechanism of environmental adaptation in sheep, but these metabolites may also play a role in methane emissions from ruminants.

## Data Availability

The datasets used and/or analyzed during the current study available from the corresponding author on reasonable request. All data generated or analyzed during this study are included in this published article.

## Supplementary Materials

The following supporting information can be downloaded at: https://www.mdpi.com/article/10.3390/ijms252010957/s1.
