# Supplementary material for: Environmental Driving of Adaptation Mechanism on Rumen Microorganisms of Sheep Based on Metagenomics and Metabolomics Data Analysis
Source: Int J Mol Sci. 2024 Oct 11;25(20):10957. doi: 10.3390/ijms252010957 (PMC11508146; doi:10.3390/ijms252010957)
Supplement: Supplementary file 1 [file ijms-25-10957-s001.zip › Table S1 Structure of rumen epithelium at different region.pdf]

Table S1 Structure of rumen epithelium at different region (μm)

	BL	ML	LP	WP	SC	SG	SS
THS	980.83 ± 105.01 *	21.33 ± 4.30	1140.67 ± 87.09	454.17 ± 39.37	59.17 ± 4.09 *	63.43 ± 2.33	46 ± 5.02
HTS	180.97 ± 11.30	360.57 ± 18.46 *	2072.10 ± 207.80 *	493.27 ± 54.87	36.37 ± 4.55	62.43 ± 4.09	48.90 ± 6.25
