# Supplementary material for: Environmental Driving of Adaptation Mechanism on Rumen Microorganisms of Sheep Based on Metagenomics and Metabolomics Data Analysis
Source: Int J Mol Sci. 2024 Oct 11;25(20):10957. doi: 10.3390/ijms252010957 (PMC11508146; doi:10.3390/ijms252010957)
Supplement: Supplementary file 1 [file ijms-25-10957-s001.zip › Table S11 The abundance of CARD Resistance.pdf]

Table S11 The abundance of CARD Resistance

**Resistance**	**THS1**	**THS2**	**THS3**	**THS4**	**THS5**	**HTS1**	**HTS2**	**HTS3**	**HTS4**	**HTS5**	**Average of THS**	**Average of HTS**
Aminocoumarin	2069	1407	1598	1592	3569	2766	1803	2392	2513	2468	2047	2388.4
Aminoglycoside	5495	2624	6469	4968	6850	3465	3226	3408	3429	4649	5281.2	3635.4
Antibacterial Free Fatty Acids	0	39	595	81	29	42	55	55	25	128	148.8	61
Bicyclomycin	127	28	383	29	939	16	0	3	0	0	301.2	3.8
Carbapenem	230	246	97	120	130	138	95	102	136	185	164.6	131.2
Cephalosporin	195	46	993	252	100	250	317	307	310	417	317.2	320.2
Cephamycin	72	16	15	172	6	40	6	81	51	20	56.2	39.6
Diaminopyrimidine	1228	569	1586	1529	1517	835	576	869	1006	916	1285.8	840.4
Disinfecting Agents And Antiseptics	990	263	1545	1127	1606	278	155	202	234	292	1106.2	232.2
Elfamycin	4	0	0	0	0	0	0	0	0	0	0.8	0
Fluoroquinolone	4937	1729	2895	3212	4316	2172	1640	1830	2023	2102	3417.8	1953.4
Fosfomycin	769	541	1735	768	2114	396	424	260	360	210	1185.4	330
Glycopeptide	17700	8953	16578	14238	19413	9774	8581	8483	9715	9703	15376.4	9251.2
Lincosamide	3469	2128	4555	2994	3849	2578	1799	2273	2524	2381	3399	2311
Macrolide	13586	9164	12689	12759	19759	9298	8124	8231	9313	9715	13591.4	8936.2
Mupirocin	4937	3008	4821	3403	4645	5270	3741	5115	5666	5504	4162.8	5059.2
Nitroimidazole	5007	4599	4821	3860	4255	5560	3670	4838	4606	3913	4508.4	4517.4
Nucleoside	55	9	0	0	0	46	7	16	32	2	12.8	20.6
Penam	16	12	66	8	447	10	0	5	5	0	109.8	4
Peptide	15299	8278	15988	14688	18826	10034	9470	9805	10549	12088	14615.8	10389.2
Phenicol	741	497	962	982	1821	650	415	605	575	577	1000.6	564.4
Pleuromutilin	2659	1201	2041	1986	2431	2362	1831	2949	2544	3110	2063.6	2559.2
Rifamycin	247	157	133	178	279	223	136	135	123	218	198.8	167

Sulfonamide	1365	378	1582	1139	1430	554	541	570	596	815	1178.8	615.2
Tetracycline	19821	11785	21969	15454	21873	14746	11361	15570	14050	14174	18180.4	13980.2
Multidrug	51061	23342	51522	41280	56022	35765	29790	35097	36513	41678	44645.4	35768.6
