# Supplementary material for: Environmental Driving of Adaptation Mechanism on Rumen Microorganisms of Sheep Based on Metagenomics and Metabolomics Data Analysis
Source: Int J Mol Sci. 2024 Oct 11;25(20):10957. doi: 10.3390/ijms252010957 (PMC11508146; doi:10.3390/ijms252010957)
Supplement: Supplementary file 1 [file ijms-25-10957-s001.zip › Table S15 Composition and nutrient levels of the basal diet.pdf]

Table S15 Composition and nutrient levels of the basal diet (DM basis).

**Items**	**Content (%)**
Silage corn	25.12
Clover	14.37
Corn	17.65
Wheat bran	9.34
Soybean meal	17.21
Brown rice	8.93
Beer yeast	3.69
Corn gluten meal	1.12
Calcium dihydrogen phosphate	0.77
Stone powder	0.89
Salt	0.46
Premix [(1)]	0.45
Total	100
**Nutrient [(2)]**	
Digestible Energy (MJ/kg)	12.28
Crude protein (%)	20.36
Neutral detergent fiber (%)	30.58
Acid detergent fiber (%)	20.11
Ether extract (%)	1.98
Calcium (%)	0.65
Total phosphorus (%)	0.59

[(1)]: The premix provided per kg of diet: $CuSO_4$ 25 mg, $FeSO_4$ H_2O 75 mg, $ZnSO_4$ H_2O 105 mg, $CoCl_2$ 0.0024 mg, Na_2SeO_3 0.016 mg, VA 12,000 IU, VD3 10,000 IU, VE 25 mg, Nicotinic acid 36 mg, Choline 1000 mg. [(2)]: Digestible energy, calcium, and total phosphorus were calculated from the composition of ingredients.
