# Supplementary material for: Environmental Driving of Adaptation Mechanism on Rumen Microorganisms of Sheep Based on Metagenomics and Metabolomics Data Analysis
Source: Int J Mol Sci. 2024 Oct 11;25(20):10957. doi: 10.3390/ijms252010957 (PMC11508146; doi:10.3390/ijms252010957)
Supplement: Supplementary file 1 [file ijms-25-10957-s001.zip › Table S2 Sample sequencing data analysis statistics.pdf]

Table S2 Sample sequencing data analysis statistics

SampleID	Clean data base(bp)	Number of Reads	GC(%)	Q20(%)	Q30(%)
THS1	7227869828	47173010	47.40	98.76	96.02
THS2	6943250642	45567618	36.74	98.43	95.13
THS3	7510404790	49388600	51.37	98.72	96.12
THS4	7065022278	46254260	49.41	98.46	95.29
THS5	7079195452	46322816	50.65	98.60	95.65
HTS1	8425097658	55541768	36.17	98.37	94.89
HTS2	6274384198	41313038	37.07	98.47	95.14
HTS3	8397305496	55425716	37.06	98.50	95.17
HTS4	7106003210	46963340	39.43	98.66	95.68
HTS5	7486129132	49313960	40.53	98.53	95.30
Average of THS	7165148598	46941261	47.11	98.59	95.64
Average of HTS	7537783939	49711564	38.05	98.51	95.24
