# Supplementary material for: Environmental Driving of Adaptation Mechanism on Rumen Microorganisms of Sheep Based on Metagenomics and Metabolomics Data Analysis
Source: Int J Mol Sci. 2024 Oct 11;25(20):10957. doi: 10.3390/ijms252010957 (PMC11508146; doi:10.3390/ijms252010957)
Supplement: Supplementary file 1 [file ijms-25-10957-s001.zip › Table S3 Sample assembly results statistics.pdf]

Table S3 Sample assembly results statistics

Sample	Contig Num.	Total Len.(bp)	Largest Len.(bp)	N50(bp)	GC(%)	Mapped(%)
THS1	191020	205658751	322055	1810	47.43	94.51
THS2	373392	301306154	214033	960	36.06	91.56
THS3	89557	106337907	342286	2066	51.09	97.63
THS4	105910	124534290	233085	2174	48.99	96.33
THS5	145527	167947394	277163	2246	47.6	93.37
HTS1	912108	579073270	144425	665	34.75	74.14
HTS2	646997	452061495	246620	765	34.72	74.68
HTS3	984276	602274079	154085	637	34.94	70.62
HTS4	825835	517138661	135104	640	38.71	75.45
HTS5	838888	518707743	159412	644	39.52	66.48
Average of THS	181081	181156899	277724	1851	46.23	94.68
Average of HTS	841620	533851049	167929	670	36.53	72.27
