# Supplementary material for: Environmental Driving of Adaptation Mechanism on Rumen Microorganisms of Sheep Based on Metagenomics and Metabolomics Data Analysis
Source: Int J Mol Sci. 2024 Oct 11;25(20):10957. doi: 10.3390/ijms252010957 (PMC11508146; doi:10.3390/ijms252010957)
Supplement: Supplementary file 1 [file ijms-25-10957-s001.zip › Table S4 Gene prediction result statistics.pdf]

Table S4 Gene prediction result statistics

Sample ID	Gene number	Total length(bp)	Average length(bp)	Max length(bp)	Min length(bp)
THS1	308868	180515616	584	24684	102
THS2	471445	256732518	544	18414	102
THS3	150267	91071942	606	18324	102
THS4	180366	106853706	592	12300	102
THS5	240688	145819638	605	20067	102
HTS1	1063879	503823972	473	27342	102
HTS2	761225	389818977	512	19290	102
HTS3	1118518	530739954	474	15990	102
HTS4	973045	456498294	469	14253	102
HTS5	988968	447800646	452	25635	102
Average of THS	270326	156198684	586	18757	102
Average of HTS	981127	465736368	476	20502	102
