# Supplementary material for: Environmental Driving of Adaptation Mechanism on Rumen Microorganisms of Sheep Based on Metagenomics and Metabolomics Data Analysis
Source: Int J Mol Sci. 2024 Oct 11;25(20):10957. doi: 10.3390/ijms252010957 (PMC11508146; doi:10.3390/ijms252010957)
Supplement: Supplementary file 1 [file ijms-25-10957-s001.zip › Table S5 Sample taxonomic rank for each species annotated to the species statistics table.pdf]

Table S5 Sample taxonomic rank for each species annotated to the species statistics table

**Sample ID**	**kingdom**	**phylum**	**class**	**order**	**family**	**genus**	**species**
THS1	5	128	123	265	561	1793	6939
THS2	5	153	137	289	665	2202	8081
THS3	4	84	88	171	355	1212	4321
THS4	4	95	94	176	377	1335	5255
THS5	5	119	118	247	525	1621	5837
HTS1	6	172	156	320	726	2537	10523
HTS2	6	164	148	299	667	2165	8328
HTS3	6	179	163	330	746	2589	10694
HTS4	6	174	156	314	720	2503	10288
HTS5	6	171	149	315	709	2459	10024
**Total**	**6**	**197**	**177**	**357**	**824**	**3396**	**17878**
