# Supplementary material for: Environmental Driving of Adaptation Mechanism on Rumen Microorganisms of Sheep Based on Metagenomics and Metabolomics Data Analysis
Source: Int J Mol Sci. 2024 Oct 11;25(20):10957. doi: 10.3390/ijms252010957 (PMC11508146; doi:10.3390/ijms252010957)
Supplement: Supplementary file 1 [file ijms-25-10957-s001.zip › Table S6 and Table S7 Phylum-level species abundance.pdf]

Table S6 Phylum-level species abundance of THS

Phylum	THS1	THS2	THS3	THS4	THS5	Average	Proportion (%)
**Bacteroidetes**	0.611194	0.214405	0.572984	0.556907	0.470770	0.485252	48.53
**Firmicutes**	0.242830	0.420459	0.303263	0.247520	0.430986	0.329012	32.90
**Proteobacteria**	0.033152	0.059645	0.076464	0.126853	0.041142	0.067451	6.75
**Chytridiomycota**	0.001159	0.032870	0.000080	0.000061	0.001268	0.007088	0.71
**Euryarchaeota**	0.010256	0.008848	0.001054	0.000842	0.001073	0.004414	0.44
**Ascomycota**	0.000833	0.023576	0.000023	0.000049	0.000901	0.005077	0.51
**Actinobacteria**	0.032729	0.033091	0.003972	0.005560	0.008505	0.016771	1.68
**Mucoromycota**	0.000774	0.019646	0.000121	0.000257	0.000879	0.004335	0.43
**Uroviricota**	0.004400	0.015765	0.004833	0.012860	0.003212	0.008214	0.82
**Chlamydiae**	0.020108	0.003586	0.003386	0.003862	0.002857	0.006760	0.68
**Others**	0.008114	0.053208	0.006334	0.016090	0.009539	0.018657	1.87
**Unclassified**	0.032768	0.099584	0.026422	0.026532	0.026610	0.042383	4.24
**Unassigned**	0.001683	0.015317	0.001063	0.002607	0.002258	0.004585	0.46
**Total**	1	1	1	1	1	1	100

Table S7 Phylum-level species abundance of HTS

Phylum	HTS1	HTS2	HTS3	HTS4	HTS5	Average	Proportion (%)
**Bacteroidetes**	0.376622	0.490807	0.481272	0.440027	0.607178	0.479181	47.92
**Firmicutes**	0.267558	0.249192	0.174302	0.275581	0.140256	0.221378	22.14
**Proteobacteria**	0.028706	0.011036	0.028085	0.019140	0.017505	0.020894	2.09
**Chytridiomycota**	0.032646	0.029615	0.034532	0.023621	0.015642	0.027211	2.72
**Euryarchaeota**	0.029774	0.006999	0.015892	0.008772	0.042470	0.020781	2.08
**Ascomycota**	0.023875	0.021929	0.025089	0.017806	0.011226	0.019985	2.00
**Actinobacteria**	0.005134	0.008522	0.005298	0.005444	0.002755	0.005431	0.54
**Mucoromycota**	0.019102	0.018055	0.020321	0.014105	0.009268	0.016170	1.62
**Uroviricota**	0.009228	0.003257	0.010806	0.006431	0.006380	0.007220	0.72
**Chlamydiae**	0.002819	0.001841	0.002117	0.003113	0.001582	0.002294	0.23
**Others**	0.057933	0.042663	0.052755	0.051453	0.041635	0.049288	4.93
**Unclassified**	0.131544	0.103083	0.134001	0.122316	0.095778	0.117344	11.7
**Unassigned**	0.015059	0.012999	0.015530	0.012191	0.008326	0.012821	1.28
**Total**	1	1	1	1	1	1	100
