# Supplementary material for: Environmental Driving of Adaptation Mechanism on Rumen Microorganisms of Sheep Based on Metagenomics and Metabolomics Data Analysis
Source: Int J Mol Sci. 2024 Oct 11;25(20):10957. doi: 10.3390/ijms252010957 (PMC11508146; doi:10.3390/ijms252010957)
Supplement: Supplementary file 1 [file ijms-25-10957-s001.zip › Table S8 and Table S9 Species-level species abundance.pdf]

Table S8 Species-level species abundance of THS

Species	THS1	THS2	THS3	THS4	THS5	Average	Proportion (%)
**Selenomonas_bovis**	0.005173	0.001172	0.092643	0.010151	0.108121	0.043452	4.35
**Prevotella_ruminicola**	0.022743	0.007749	0.004798	0.007834	0.003649	0.009354	0.94
**Prevotella_multisaccharivorax**	0.022651	0.010755	0.045133	0.060699	0.044864	0.036820	3.68
**Prevotella_sp._AGR2160**	0.025781	0.006195	0.045042	0.039212	0.037838	0.030814	3.08
**Selenomonas_ruminantium**	0.014468	0.035354	0.025784	0.003526	0.027288	0.021284	2.13
**Prevotella_sp._ne3005**	0.015134	0.004188	0.001574	0.000815	0.001610	0.004664	0.47
**Succiniclasticum_ruminis**	0.024594	0.014214	0.002625	0.000176	0.017831	0.011888	1.19
**Dialister_succinatiphilus**	0.000177	0.002681	0.024889	0.047965	0.039887	0.023120	2.31
**Prevotella_sp._tc2_28**	0.007926	0.006358	0.003239	0.003163	0.003113	0.004760	0.48
**Prevotella_sp._tf2_5**	0.003580	0.022332	0.001002	0.000595	0.003969	0.006296	0.63
**Others**	0.580136	0.578185	0.470004	0.552584	0.439599	0.524101	52.41
**Unclassified**	0.275955	0.295501	0.282204	0.270675	0.269974	0.278862	27.89
**Unassigned**	0.001683	0.015317	0.001063	0.002607	0.002258	0.004585	0.46
**Total**	1	1	1	1	1	1	100

Table S9 Species-level species abundance of HTS

Species	HTS1	HTS2	HTS3	HTS4	HTS5	Average	Proportion (%)
**Selenomonas_bovis**	0.000938	0.000035	0.000066	0.000043	0.000033	0.000223	0.02
**Prevotella_ruminicola**	0.025912	0.033316	0.031037	0.021428	0.035503	0.029439	2.94
**Prevotella_multisaccharivorax**	0.000249	0.000396	0.000298	0.000287	0.000311	0.000308	0.03
**Prevotella_sp._AGR2160**	0.000470	0.000813	0.000645	0.000686	0.000885	0.000700	0.07
**Selenomonas_ruminantium**	0.018691	0.002065	0.003509	0.001790	0.001311	0.005473	0.55
**Prevotella_sp._ne3005**	0.012821	0.025706	0.016820	0.013547	0.028354	0.019449	1.94
**Succiniclasticum_ruminis**	0.013172	0.012371	0.012380	0.009198	0.013930	0.012210	1.22
**Dialister_succinatiphilus**	0.000033	0.000015	0.000017	0.000014	0.000019	0.000019	0.00
**Prevotella_sp._tc2_28**	0.013549	0.016676	0.017895	0.007756	0.025121	0.016199	1.62
**Prevotella_sp._tf2_5**	0.009836	0.018414	0.009590	0.008476	0.023193	0.013902	1.39
**Others**	0.533638	0.505276	0.501083	0.555107	0.465043	0.512029	51.20
**Unclassified**	0.355632	0.371919	0.391130	0.369476	0.397974	0.377226	37.72
**Unassigned**	0.015059	0.012999	0.015530	0.012191	0.008326	0.012821	1.28
**Total**	1	1	1	1	1	1	100
