# Supplementary figures and images for: Environmental Driving of Adaptation Mechanism on Rumen Microorganisms of Sheep Based on Metagenomics and Metabolomics Data Analysis
Source: Int J Mol Sci. 2024 Oct 11;25(20):10957. doi: 10.3390/ijms252010957 (PMC11508146; doi:10.3390/ijms252010957)

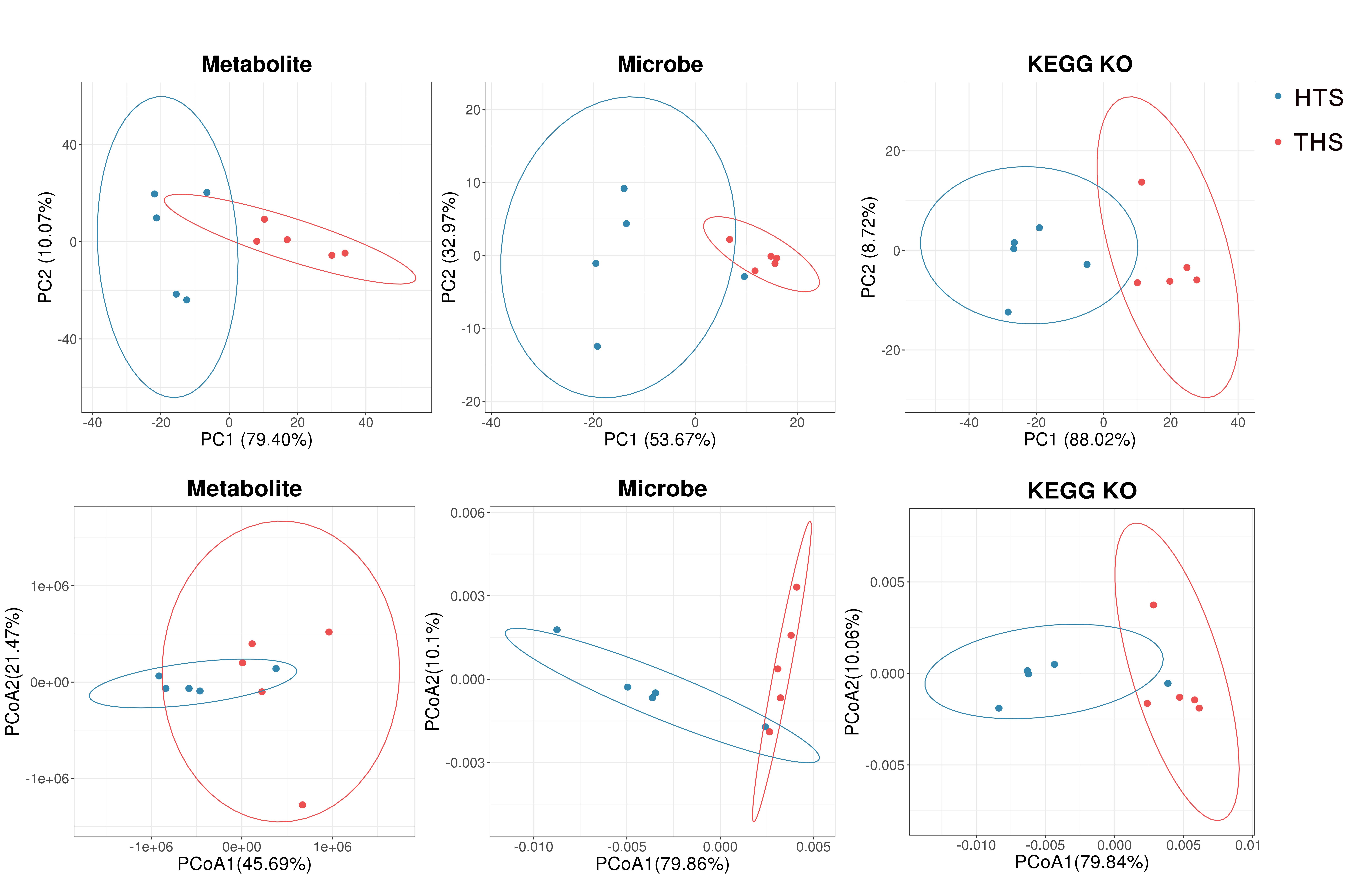

Supplement: Supplementary file 1 [file ijms-25-10957-s001.zip › Figure S2 PCA.png]
